# Risk factors for cancer of the oral cavity and oro-pharynx in Cuba

**DOI:** 10.1054/bjoc.2000.1825

**Published:** 2001-07

**Authors:** L Fernandez Garrote, R Herrero, R M Ortiz Reyes, S Vaccarella, J Lence Anta, L Ferbeye, N Muñoz, S Franceschi

**Affiliations:** Instituto de Oncologia y Radiobiologia, Calle 29 y E Vedado, La Habana, 10400, Cuba; International Agency for Research on Cancer, 150 Cours Albert Thomas, F-69372 Lyon cédex 08, France; Now at Costa Rica Cancer Institute, San José, Costa Rica; Servizio di Epidemiologia e biostatistica, IRCSS Centro di Riferimento Oncologico di Aviano, Via Pedemontana occidentale 12, I-33081 Aviano (PN), Italy

**Keywords:** oral cancer, alcohol, tobacco, sexual habits

## Abstract

In terms of worldwide levels, Cuba has an intermediate incidence of cancer of the oral cavity and oro-pharynx. We studied 200 cases of cancer of the oral cavity and pharynx, of whom 57 women (median age = 64) and 200 hospital controls, frequency matched with cases by age and sex, in relation to smoking and drinking history, intake of 25 foods or food groups, indicators of oral hygiene and sexual activity, and history of sexually transmitted diseases. Odds ratios (OR) and 95% confidence intervals (CI) were obtained from unconditional multiple logistic regressions and adjusted for age, sex, area of residence, education, and smoking and drinking habits. In the multivariate model, high educational level and white-collar occupation, but not white race, were associated with halving of oral cancer risk. Smoking ≥30 cigarettes per day showed an OR of 20.8 (95% CI: 8.9–48.3), similar to smoking ≥4 cigars daily (OR = 20.5). Drinking ≥ 70 alcoholic drinks per week showed an OR of 5.7 (95% CI: 1.8–18.5). Hard liquors were by far the largest source of alcohol. Increased risk was associated with the highest tertile of intake for maize (OR = 1.9), meat (OR = 2.2) and ham and salami (OR = 2.0), whereas high fruit intake was associated with significantly decreased risk (OR = 0.4). Among indicators of dental care, number of missing teeth and poor general oral condition at oral inspection showed ORs of 2.7 and 2.6, respectively. Number of sexual partners, marriages or contacts with prostitutes, practice of oral sex and history of various sexually transmitted diseases, including genital warts, were not associated with oral cancer risk. 82% of oral cancer cases in Cuba were attributable to tobacco smoking, 19% to smoking cigars or pipe only. The fractions attributable to alcohol drinking (7%) and low fruit intake (11%) were more modest. Thus, decreases in cigarette and cigar smoking are at present the key to oral cancer prevention in Cuba. © 2001 Cancer Research Campaign http://www.bjcancer.com


*British Journal of Cancer* (2001) **85**(1), 46–54
© 2001 Cancer Research Campaign
doi: 10.1054/ bjoc.2001.1825, available online at http://www.idealibrary.com on IDEAL®

http://www.bjcancer.com

### Risk factors for cancer of the oral cavity and oro-pharynx in Cuba

**L Fernandez Garrote[1], R Herrero[2,3], RM Ortiz Reyes[1], S Vaccarella[4], J Lence Anta[1], L Ferbeye[1], N Muñoz[2] and S Franceschi[2]**

[1]Instituto de Oncologia y Radiobiologia, Calle 29 y E Vedado, La Habana 10400, Cuba; [2]International Agency for Research on Cancer, 150, Cours Albert Thomas, F-69372 Lyon cédex 08, France; [3]Now at Costa Rica Cancer Institute, San José, Costa Rica; [4]Servizio di Epidemiologia e biostatistica, IRCSS Centro di Riferimento Oncologico di Aviano, Via Pedemontana occidentale 12, I-33081 Aviano (PN), Italy

**Summary** In terms of worldwide levels, Cuba has an intermediate incidence of cancer of the oral cavity and oro-pharynx. We studied 200 cases of cancer of the oral cavity and pharynx, of whom 57 women (median age = 64) and 200 hospital controls, frequency matched with cases by age and sex, in relation to smoking and drinking history, intake of 25 foods or food groups, indicators of oral hygiene and sexual activity, and history of sexually transmitted diseases. Odds ratios (OR) and 95% confidence intervals (CI) were obtained from unconditional multiple logistic regressions and adjusted for age, sex, area of residence, education, and smoking and drinking habits. In the multivariate model, high educational level and white-collar occupation, but not white race, were associated with halving of oral cancer risk. Smoking ≥30 cigarettes per day showed an OR of 20.8 (95% CI: 8.9–48.3), similar to smoking ≥4 cigars daily (OR = 20.5). Drinking ≥ 70 alcoholic drinks per week showed an OR of 5.7 (95% CI: 1.8–18.5). Hard liquors were by far the largest source of alcohol. Increased risk was associated with the highest tertile of intake for maize (OR = 1.9), meat (OR = 2.2) and ham and salami (OR = 2.0), whereas high fruit intake was associated with significantly decreased risk (OR = 0.4). Among indicators of dental care, number of missing teeth and poor general oral condition at oral inspection showed ORs of 2.7 and 2.6, respectively. Number of sexual partners, marriages or contacts with prostitutes, practice of oral sex and history of various sexually transmitted diseases, including genital warts, were not associated with oral cancer risk. 82% of oral cancer cases in Cuba were attributable to tobacco smoking, 19% to smoking cigars or pipe only. The fractions attributable to alcohol drinking (7%) and low fruit intake (11%) were more modest. Thus, decreases in cigarette and cigar smoking are at present the key to oral cancer prevention in Cuba. © 2001 Cancer Research Campaign http://www.bjcancer.com

**Keywords**: oral cancer; alcohol; tobacco; sexual habits

Incidence rates for cancer of the oral cavity and oro-pharynx (WHO, 1997) in Cuba, standardized to the world standard population, were in 1986 7.2 and 2.9 per 100 000 in males and females, respectively (Parkin et al, 1992). The Cuban population, therefore, shows rates which are similar to those in whites in the United States, but approximately 50% lower than in high-risk areas in Latin America (e.g., Puerto Rico, Brazil, etc.) (Franceschi et al, 2000). Oral cancer has been stable in both sexes between 1980 and 1998 (Fernandez Garrote, personal communication), as in most North and South American countries, but at variance with the upward trends observed in several areas of Europe in the same period (Franceschi et al, 2000).

Over 80% of oral cancers in the United States (Blot et al, 1988) and in Europe (Negri et al, 1993) are attributable to tobacco smoking and heavy alcohol consumption, with low intake of fresh fruit and vegetables playing a smaller role. Little is known on the relative importance of various risk factors on oral cancer aetiology in Cuba though certain features of the Cuban population, including heavy cigarette and cigar smoking (Corrao et al, 2000) and recent dietary deficiencies offer special research opportunities.

We herein report on the findings of the first case–control study on cancer of the oral cavity and oro-pharynx in Cuba where, in addition to smoking, alcohol drinking, and dietary habits, the potential contribution of oral hygiene, dentition, sexual habits and sexually transmitted diseases has been examined.

*Received 10 January 2001*
*Revised 12 January 2001*
*Accepted 21 February 2001*

*Correspondence to:* S Franceschi

### MATERIALS AND METHODS

The present Cuban case–control study was part of an international study of oral cancer and HPV carried out in Italy (Talamini et al, 2000), Spain, Northern Ireland, Poland, Canada, India, Sudan and Australia, and coordinated by the International Agency for Research on Cancer (Herrero et al, 2000).

Between April 1996 and July 1999 first incident cases of cancer of the oral cavity and oro-pharynx, prior to any cancer treatment, were identified in the Instituto Nacional de Oncologia y Radiobiologia (INOR) of Cuba in the City of Havana. Of 227 potential cases, one refused and 26 were too sick to be interviewed. A total of 200 cases (median age = 64; range 28–91 years), including 57 women, were enrolled. In 153 of these, cancer involved the mouth only, in 19 the oro-pharynx only, and in 28 both sites. The distribution by stage was as follows: stage 1: 17%; stage 2: 19%; stage 3: 19% and stage 4: 44%. All cases had their interview and oral examination before any cancer treatment.

Eligible controls had been admitted to INOR and 3 other major hospitals in Havana City for diseases unrelated to smoking or drinking habits. They were frequency-matched with cases by age and gender. Out of 253 identified subjects, 48 refused and 5 were too sick to be interviewed. Controls were therefore 200 subjects (median age = 62; range 25–88 years), including 64 women. 24% had been admitted for acute surgical diseases; 18% for trauma or orthopaedic conditions; 8% for acute medical conditions; 8% for skin diseases; 7% for neoplastic diseases; 11% for eye diseases; and 17% for other miscellaneous diseases. Cases and controls were interviewed during their hospital stay by one trained dentist (MRO). Questions included smoking status (never, ex- or current smokers), daily number of cigarettes/cigars and grams of pipe tobacco, age at starting and duration of the habit. One pipeful of regular pipe tobacco corresponded to one cigar.

The frequency of consumption of the commonest alcoholic beverages was investigated. Taking into account the different ethanol concentration, one drink corresponded to approximately 40 ml of hard liquor, 330 ml of beer, and 125 ml of wine corresponding to 12–13 g of ethanol. Never smokers and never drinkers were individuals who had abstained from any alcoholic beverage lifelong. Ex-drinkers and ex-smokers had abstained from any type of drinking or smoking for at least 12 months.

The questionnaire included information on socio-demographic characteristics, prior occurrence of sexually transmitted and other infections, cancer family history, and a dietary questionnaire. In the early 1990s, substantial changes in eating and drinking habits of the Cuban population were caused by reduction in the availability of meat, dairy products, fruit and vegetables. We attempted to elicit from patients details of their life-time dietary habits, rather than recent diet. Indicators of oral hygiene were self-reported by means of 9 specific questions. The number of missing teeth which had not been replaced and the general oral condition, on the basis of presence of tartar, decayed teeth, and mucosal irritation, were evaluated by the interviewing dentist through inspection of the mouth. The interviewer was aware of case/control status.

Odds ratios (ORs) and corresponding 95% confidence intervals (CIs) were computed using unconditional multiple logistic regression models. All models included terms for age quinquennium, gender, area of residence (Havana City and surrounding areas) educational years, and smoking and drinking habits, in addition to other variables as specified. Approximate intake tertiles were computed for food groups. Foods were also analysed as 4 major groups, i.e., starchy foods (including cereal dishes and pulses); animal foods (including meats, fish, and cheese); all vegetables; and all fruit. Attributable risk fractions were computed according to a method which implies knowledge of the risk estimates and of the joint distribution of risk factors among cases only, and is therefore applicable to hospital-based case–control studies (Mezzetti et al, 1996).

### RESULTS

Cases of oral cancer and controls were by study design similar in respect to age and gender (Table 1). After allowance for area of

**Table 1** Distribution of 200 cases of the oral cavity and oropharynx and 200 controls and corresponding odds ratios (OR) and 95% confidence intervals (CI) by selected socio-demographic variables. [a]Cuba, 1996–1999

| | Cases (*n*) | Controls (*n*) | OR[b] | | (95% CI) |
|---|---|---|---|---|---|
| Age (years) | | | | | |
| <55 | 40 | 64 | – | | |
| 55–64 | 56 | 49 | – | | |
| 65–74 | 66 | 56 | – | | |
| ≥75 | 38 | 31 | – | | |
| Gender | | | | | |
| Males | 143 | 136 | – | | |
| Females | 57 | 64 | – | | |
| Ethnic group | | | | | |
| White | 161 | 135 | 1[c] | | |
| Black | 22 | 29 | 0.69 | | (0.34–1.39) |
| Mulatto | 17 | 35 | 0.42 | | (0.20–0.87) |
| Education (years) | | | | | |
| ≥9 | 65 | 113 | 1[c] | | |
| 6–8 | 66 | 44 | 2.10 | | (1.17–3.76) |
| <6 | 69 | 43 | 2.05 | | (1.09–3.87) |
| $\chi^2_1$ for trend | | | | 5.52; $P = 0.02$ | |
| Spouse's education (years) | | | | | |
| ≥9 | 61 | 118 | 1[c] | | |
| 6–8 | 61 | 39 | 1.95 | | (1.05–3.62) |
| <6 | 78 | 43 | 2.52 | | (1.37–4.64) |
| $\chi^2_1$ for trend | | | | 8.89; $P < 0.01$ | |
| Number of siblings | | | | | |
| <4 | 57 | 62 | 1[c] | | |
| 4–7 | 76 | 76 | 0.91 | | (0.52–1.61) |
| ≥8 | 67 | 62 | 0.79 | | (0.43–1.44) |
| $\chi^2_1$ for trend | | | | 0.62; $P = 0.43$ | |
| Occupation | | | | | |
| White collar | 51 | 85 | 1[c] | | |
| Blue collar | 68 | 57 | 1.73 | | (0.97–3.09) |
| Farmers | 37 | 19 | 2.22 | | (0.97–5.10) |
| Housewives and other | 35 | 30 | 1.94 | | (0.87–4.33) |

[a]Some strata do not add up to the total because of missing values. [b]Estimates from unconditional logistic regression equations, including terms for gender, age, area of residence, education, smoking and drinking habits. [c]Reference category.

© 2001 Cancer Research Campaign 

residence, education, and smoking and drinking habits, blacks (OR = 0.7) and mulattos (OR = 0.4) showed a somewhat lower oral cancer risk than whites. Individuals who reported fewer than 6 years of education showed an OR of 2.1 (95% CI: 1.1–3.9) compared to those who reported 9 years or more. Spouse's education also seemed influential, whereas number of siblings, as a proxy of general living in childhood, was unrelated to oral cancer risk. Among major occupational groups, blue collar workers (OR = 1.7) and farmers (OR = 2.2) showed increases of borderline significance in cancer risk compared to white collar workers.

Heavy smoking was very common among cases, with 32% reporting smoking 30 cigarettes or more per day (Table 2). The OR for smoking 30 cigarettes per day or more, compared to never smokers, was 20.8 (95% CI: 8.9–48.3) among current smokers. Former smokers showed an OR of 6.3 (95% CI: 3.0–13.4). 9% of cases and 5% of controls smoked cigars, but not cigarettes; among these, 6 of cases and 1 of controls smoked pipe, in addition to cigars, one control used pipe only. Smoking of cigars or pipe only was associated with an OR of 4.3 for 3 or fewer and 20.5 for 4 cigars or equivalents per day or more. Long-duration smoking and early starting smoking were associated with especially increased risks (Table 2). Smoking cessation was followed by a significant risk reduction only 10 years or more after quitting (OR compared to persistent smokers = 0.4; 95% CI: 0.2–0.9). ORs in former smokers were not modified by adjustment for intensity and duration of smoking. The vast majority of smokers in Cuba had smoked only black tobacco (85% of cases and 80% of control subjects) and cigarettes without a filter (94% of cases and 88% of control subjects).

Approximately half of the study population did not report drinking alcoholic beverages, and only 21% of cases and 10% of controls drank 21 or more drinks per week (ORs for 21–69 = 2.2, and ≥ 70 drinks/week = 5.7; Table 3). Among current drinkers, duration of the habit and age at starting were not associated significantly with risk. Among former drinkers, the OR declined steadily after stopping and it was 0.3 (95% Cl: 0.1–0.8) 10 or more years after drinking cessation. Hard liquors were consumed in larger amounts than beer or wine and they were associated with an OR of 5.1 for 70 or more drinks per week, after allowance for the intake of beer and wine. At equal intake levels, the effect of the 3 major types of alcoholic beverages considered did not differ appreciably (Table 3).

Table 4 shows the combined effect of smoking and drinking on oral cancer risk. Drinking among non-smokers or former smokers seemed unrelated to oral cancer risk. At the highest levels of both exposures (i.e., ≥ 30 cigarettes per day and ≥ 21 drinks per week) the OR was 111 (95% CI: 22.7–543.7), which is compatible with a multiplicative or supra-multiplicative effect of the 2 habits. Drinking cessation did not seem to confer any benefit if smoking was continued (OR = 33.6 among former drinkers who smoked ≥ 30 cigarettes/day).

Oral cancer risk by intake tertile of 25 foods or food groups is shown in Table 5: significant direct associations emerged for maize dishes (OR in the highest vs lowest tertile = 1.9; 95% CI: 1.0–3.8), meat (OR = 2.2; 95% CI: 1.2–3.9) and ham and salami (OR = 2.0; 95% CI: 1.1–3.7). High intake tertile of most starchy foods and animal foods showed ORs above 1 whereas a high

**Table 2** Odds ratios (OR) and corresponding 95% confidence intervals (CI) for cancer of the oral cavity and oro-pharynx by smoking habits: 200 cases and 200 controls[a]. Cuba, 1996–1999

| | Cases (*n*) | Controls (*n*) | OR[b] | | (95% CI) |
|---|---|---|---|---|---|
| Smoking habit | | | | | |
| Never smokers | 16 | 81 | 1[c] | | |
| Former smokers | 42 | 39 | 6.31 | | (2.97–13.43) |
| Current smokers | | | | | |
| <20 cigarettes/day | 25 | 25 | 6.31 | | (2.75–14.46) |
| 20–29 cigarettes/day | 36 | 26 | 9.63 | | (4.28–21.64) |
| ≥ 30 cigarettes/day[d] | 63 | 18 | 20.77 | | (8.93–48.30) |
| $\chi^2_1$ for trend | | | 45.91; $P < 0.001$ | | |
| < 4 cigars or equiv./day | 6 | 7 | 4.31 | | (1.13–16.38) |
| ≥ 4 cigars or equiv./day | 11 | 3[e] | 20.45 | | (4.67–89.65) |
| $\chi^2_1$ for trend | | | 8.23 $P < 0.01$ | | |
| Age started smoking (yrs) | | | | | |
| ≥17 | 45 | 29 | 1[c] | | |
| 14–16 | 42 | 23 | 3.24 | | (1.69–6.20) |
| <14 | 54 | 27 | 2.76 | | (1.55–4.89) |
| $\chi^2_1$ for trend | | | | 0.16; $P = 0.70$ | |
| Duration of smoking (yrs) | | | | | |
| <38 | 26 | 29 | 1[c] | | |
| 38–50 | 53 | 23 | 3.78 | | (2.05–6.99) |
| ≥51 | 62 | 27 | 3.39 | | (1.82–6.32) |
| $\chi^2_1$ for trend | | | | 0.53; $P = 0.47$ | |
| Years since quit smoking | | | | | |
| Current smoker | 142 | 80 | 1[c] | | |
| <10 | 28 | 14 | 2.32 | | (1.11–4.81) |
| ≥10 | 11 | 25 | 0.37 | | (0.16–0.85) |
| $\chi^2_1$ for trend | | | | 9.80; $P < 0.01$ | |

[a]Some strata do not add up to the total because of missing values. [b]Estimates from unconditional logistic regression equations including terms for gender, age, area of residence, education and smoking and drinking habits [c]Reference category. [d]It includes 25 cigar smokers who also smoked any number of cigarettes and cigars. [e]It includes 1 who smoked pipe only.

 © 2001 Cancer Research Campaign

**Table 3** Odds ratios (OR) and corresponding 95% confidence interval (CI) for cancer of the oral cavity and oro-pharynx by drinking habit: 200 cases and 200 controls.[a] Cuba, 1996–1999

| | Cases (*n*) | Controls (*n*) | OR[c] | (95% CI) | OR[e] | (95% CI) |
|---|---|---|---|---|---|---|
| Drinking habit | | | | | | |
| Abstainers | 83 | 106 | 1[d] | | | |
| Former drinkers | 36 | 34 | 1.04 | (0.52–2.06) | | |
| Current drinkers (drinks/wk)[b] | | | | | | |
| <7 | 15 | 21 | 1.09 | (0.46–2.57) | | |
| 7–20 | 25 | 19 | 1.60 | (0.70–3.67) | | |
| 21–69 | 21 | 14 | 2.20 | (0.89–5.45) | | |
| ≥70 | 20 | 6 | 5.73 | (1.77–18.52) | | |
| $\chi^2_1$ for trend | | | 8.75; *P* < 0.01 | | | |
| Age at start drinking (yrs) | | | | | | |
| ≥21 | 20 | 9 | 1[d] | | | |
| 17–20 | 28 | 28 | 0.86 | (0.43–1.71) | | |
| <17 | 33 | 23 | 0.42 | (0.16–1.08) | | |
| $\chi^2_1$ for trend | | | 1.30; *P* = 0.26 | | | |
| Duration of drinking (yrs) | | | | | | |
| <33 | 20 | 22 | 1[d] | | | |
| 33–44 | 31 | 21 | 1.98 | (0.93–4.22) | | |
| ≥45 | 30 | 17 | 1.81 | (0.85–3.87) | | |
| $\chi^2_1$ for trend | | | 0.56; *P* = 0.46 | | | |
| Years since quit drinking | | | | | | |
| Current | 81 | 60 | 1[d] | | | |
| <10 | 21 | 18 | 0.74 | (0.31–1.80) | | |
| ≥10 | 14 | 16 | 0.28 | (0.10–0.80) | | |
| $\chi^2_1$ for trend | | | 5.00; *P* = 0.03 | | | |
| Hard liquor (drinks/wk)[b] | | | | | | |
| 0 | 86 | 109 | 1[d] | | 1[d] | |
| 1–7 | 19 | 17 | 1.64 | (0.72–3.75) | 1.25 | (0.47–3.33) |
| 8–20 | 25 | 26 | 1.26 | (0.60–2.63) | 0.98 | (0.40–2.40) |
| 21–69 | 15 | 4 | 5.23 | (1.47–18.62) | 4.18 | (1.05–16.54) |
| ≥70 | 15 | 3 | 6.36 | (1.59–25.53) | 5.14 | (1.13–23.34) |
| $\chi^2_1$ for trend | | | 9.01; *P* < 0.01 | | 4.58; *P* < 0.05 | |
| Beer (drinks/wk)[b] | | | | | | |
| 0 | 98 | 120 | 1[d] | | 1[d] | |
| <7 | 36 | 29 | 1.66 | (0.86–3.20) | 1.54 | (0.61–3.88) |
| ≥7 | 29 | 17 | 2.26 | (1.05–4.88) | 1.52 | (0.51–4.55) |
| $\chi^2_1$ for trend | | | 4.31; *P* < 0.05 | | 0.85; *P* = 0.36 | |
| Wine (drinks/wk)[b] | | | | | | |
| 0 | 129 | 141 | 1[d] | | 1[d] | |
| <2 | 26 | 19 | 1.55 | (0.74–3.26) | 0.96 | (0.38–2.44) |
| ≥2 | 9 | 6 | 1.35 | (0.40–4.55) | 0.75 | (0.18–3.19) |
| $\chi^2_1$ for trend | | | 0.57; *P* = 0.46 | | | 0.15; *P* = 0.70 |

[a]Some strata do not add up to the total because of missing value. [b]One drink corresponds to approximately 125 ml of wine, 330 ml of beer, and 40 ml of liquor (i.e., 12–13 g of ethanol). [c]Estimates from unconditional logistic regression equations, including terms for gender, age, area of residence, education and smoking and drinking habits. [d]Reference category. [e]As in footnote [c]plus all listed beverages.

**Table 4** Odds ratios (OR) and corresponding 95% confidence intervals (CI)[a,b] of oral cavity and oropharynx cancer according to various combinations of smoking and drinking habits: 200 cases and 200 controls. Cuba, 1996–1999

| | Alcohol intake (drinks/week) | | | |
|---|---|---|---|---|
| Smoking habit | 0 | <21 | 21+ | Ex drinkers[d] |
| Never smokers | 14/58 | 1/11 | 0/3 | 1/9 |
| OR | 1[c] | 0.53 | | 1[c] |
| (95% CI) | | (0.06–4.73) | | |
| Current smokers (N/day) | | | | |
| 1–29 | 35/27 | 17/18 | 15/7 | 11/9 |
| OR | 6.64 | 10.98 | 26.72 | 13.13 |
| (95% CI) | (2.80–15.73) | (3.68–32.79) | (7.15–99.94) | (0.61–281.57) |
| ≥30 | 15/5 | 15/3 | 21/3 | 12/7 |
| OR | 10.49 | 42.31 | 111.16 | 33.63 |
| (95% CI) | (2.88–38.15) | (8.43–212.32) | (22.73–543.73) | (1.55–728.88) |
| Former smokers[e] | 19/16 | 7/8 | 4/6 | 12/9 |
| OR | 1[c] | 0.89 | 0.70 | 7.74[f] |
| (95% CI) | | (0.23–3.48) | (0.14–3.59) | (1.42–42.29) |

[a]Some strata do not add up to the total because of missing value. [b]Estimates from unconditional logistic regression equations, including terms for gender age, area of residence and education. [c]Reference category. [d]Alcohol-adjusted. [e]Smoking-adjusted. [f]As compared to never smoking never drinkers.

© 2001 Cancer Research Campaign 

**Table 5** Odds ratios (OR) and corresponding 95% confidence intervals (CI) for cancer of the oral cavity and oro-pharynx according to approximate intake tertile of 25 foods or foodgroups[a]: 200 cases and 200 controls. Cuba, 1996–1999

| | | Approximate intake tertile | | |
|---|---|---|---|---|
| | | OR (95% CI)[b] | | $\chi^2_1$ for trend |
| | Servings/week | II | III (highest) | |
| Food | | | | |
| Milk | (<1; 1–6; ≥7) | 0.72(0.33–1.61) | 0.72(0.39–1.33) | 0.95; $P = 0.33$ |
| Yoghurt | (0; <2; ≥2) | 0.74(0.42–1.32) | 1.16(0.65–2.08) | 0.26; $P = 0.61$ |
| Bread | (<7; 7; ≥8) | 0.94(0.49–1.81) | 2.67(0.55–12.99) | 0.22; $P = 0.64$ |
| Pasta or rice | (<7; 7; ≥8) | 0.83(0.33–2.08) | 1.43(0.36–5.70) | 0.14; $P = 0.71$ |
| Maize dishes | (0; <2; ≥2) | 1.00(0.53–1.91) | 1.94(1.00–3.79) | 5.39; $P < 0.05$ |
| Meat | (<3; 3–5; ≥6) | 1.11(0.62–1.98) | 2.18(1.21–3.91) | 6.78; $P < 0.01$ |
| Fish | (<2; 2–3; ≥4) | 0.77(0.44–1.34) | 1.49(0.82–2.73) | 1.41; $P = 0.23$ |
| Ham and salami | (0; <2; ≥2) | 0.94(0.51–1.72) | 2.03(1.11–3.74) | 6.23; $P < 0.02$ |
| Eggs | (<3; 3–5; ≥6) | 1.55(0.87–2.75) | 1.64(0.91–2.98) | 2.55; $P = 0.11$ |
| Cheese | (<1; 1–3; ≥4) | 1.07(0.62–1.83) | 1.85(1.01–3.38) | 3.49; $P = 0.06$ |
| Potatoes | (<3; 3–4; ≥5) | 0.88(0.49–1.58) | 0.95(0.54–1.67) | 0.03; $P = 0.87$ |
| Raw green vegetables | (<2; 2–5; ≥6) | 1.12(0.63–2.02) | 0.89(0.50–1.59) | 0.21; $P = 0.65$ |
| Cruciferae | (0; <4; ≥4) | 1.70(0.99–2.93) | 1.11(0.62–2.01) | 0.37; $P = 0.54$ |
| Carrots | (0; <3; ≥3) | 0.61(0.34–1.11) | 0.86(0.49–1.52) | 0.48; $P = 0.49$ |
| Tomatoes | (<4; 4–6; ≥7) | 0.71(0.37–1.35) | 0.63(0.36–1.12) | 2.27; $P = 0.13$ |
| Pulses | (<4; 4–6; ≥7) | 0.96(0.46–1.99) | 1.49(0.81–2.72) | 2.14; $P = 0.14$ |
| Fruit juices | (<2; 2–4; 4) | 0.71(0.41–1.24) | 0.77(0.42–1.40) | 0.79; $P = 0.37$ |
| Apples or pears | (0; ≥1) | 1.49(0.53–4.18) | | |
| Citrus fruit | (<2; 2–4; ≥5) | 0.85(0.47–1.55) | 0.78(0.42–1.45) | 0.59; $P = 0.44$ |
| Bananas | (<3; 3–5; ≥6) | 0.64(0.37–1.10) | 0.62(0.34–1.13) | 2.65 $P = 0.10$ |
| Cakes and desserts | (<1; 1–4; ≥5) | 0.72(0.40–1.29) | 1.00(0.56–1.77) | 0.00; $P = 0.99$ |
| Major food groups[c] | | | | |
| Starchy foods | (<16, 16–19, > 19) | 0.77(0.42–1.42) | 1.91(0.90–4.05) | 2.50; $P = 0.11$ |
| Animal foods | (<9, 9–15, > 15) | 1.04(0.56–1.93) | 2.00(0.96–4.18) | 3.12; $P = 0.08$ |
| Vegetables | (<12, 12–19, >19) | 0.67(0.36–1.23) | 0.78(0.40–1.51) | 0.48; $P = 0.49$ |
| Fruits | (<7, 7–13, >13) | 0.71(0.39–1.29) | 0.43(0.21–0.89) | 5.05; $P < 0.05$ |

[a]Reference category was at the lowest tertile. [b]Estimates from unconditional logistic regression equations, including terms for gender, age, area of residence, education, smoking and drinking habits. [c]Adjusted as in footnote 2, plus all 4 major food groups.

intake of most vegetables and fruits generally showed ORs below 1. In order to evaluate the dietary pattern, 4 major food groups were considered jointly. After allowance for the other 3 major food groups, high intake of fruit was associated with an OR of 0.4 (95% CI: 0.2–0.9) (Table 5).

Cases reported that they brushed their teeth less often, but had gum bleeding more frequently than controls. However, after allowing for smoking and drinking habits, differences were not significant (Table 6). Use of mouth wash twice per week or more was associated with a significant OR of 0.3. 67% of cases and 63% of controls reported wearing a removable denture. Neither ever nor long-duration (≥ 10 years) use of a denture or dental check-ups had a clear influence on oral cancer risk. At visual inspection of the mouth, the number of missing teeth was significantly higher among oral cancer cases compared to control subjects (OR for ≥ 16 missing teeth = 2.7; 95% CI: 1.2–6.1). After allowance for education, smoking and drinking habits, poor general oral condition was 2.6-fold (95% CI: 1.2–5.2) more frequent among cases than control subjects (Table 6).

Cases and controls reported with similar frequency a history of warts at various body sites (Table 7). A history of genital warts (1 case and 2 controls), herpetic lesions, candidiasis or gonorrhoea was non-significantly less frequent among oral cancer cases than control subjects. No cases, but 8 controls reported a history of syphilis. A downward trend in risk was found according to number of lifetime sexual partners (OR for ≥ 11 vs ≤ 1 partner = 0.5), of marriages (OR for ≥ 3 vs ≤ 1 = 0.6), and of sexual partners who were prostitutes (OR = ≥ 6 prostitutes vs 0 = 0.5, men only, $P < 0.05$). Age at first marriage, and occasional (OR = 0.6) or frequent (OR = 0.8) practice of oral sex did not seem to affect oral cancer risk (Table 7). No cases and 2 control subjects reported homosexual intercourse.

All variables were re-examined separately in men and women for cancers of the oral cavity and oro-pharynx, yielding similar risk patterns.

### DISCUSSION

Tobacco smoking is by far the predominant cause of oral cancer in Cuba. In the present study, the risk percentage attributable to smoking alone was 82% (95% Cl: 72–91%). The percentage was higher in men (85%) than women (72%). Alcohol drinking and low fruit intake accounted for 7% and 11% respectively of oral cancer, but confidence intervals of the attributable percentages were broad (–20 and 34; and –4 and 25, respectively). The relative rarity of cancer of the oro-pharynx (10% of the present case series) is in agreement with the modest importance of alcohol intake in Cuba, since in populations where the risk fraction attributable to alcohol intake is high (e.g., Northern France or Northern Italy; IARC, 1988) cancer of the pharynx is approximately as frequent as oral cancer (Franceschi et al, 2000).

The impact of tobacco smoking can be appreciated better considering that, despite the high proportion of heavy smokers we found in the 1990s, tobacco consumption in Cuba had declined

 © 2001 Cancer Research Campaign

**Table 6** Odds ratios (OR) and corresponding 95% confidence intervals (CI) for cancer of the oral cavity and oro-pharynx according to indicators of oral hygiene and dentition: 200 cases and 200 controls[a]. Cuba, 1996–1999

| | Cases (*n*) | Controls (*n*) | OR[b] | (95% CI) |
|---|---|---|---|---|
| *Self-reported*: | | | | |
| Tooth brushing (times/day) | | | | |
| ≥2 | 105 | 143 | 1[c] | |
| 1 | 25 | 15 | 1.17 | (0.52–2.66) |
| <1 | 21 | 13 | 1.94 | (0.83–4.50) |
| $\chi^2_1$ for trend | | | 2.26; $P = 0.13$ | |
| Gum bleeding | | | | |
| Never | 109 | 101 | 1[c] | |
| Sometimes | 31 | 62 | 0.49 | (0.27–0.89) |
| Always/almost always | 10 | 8 | 2.03 | (0.66–6.25) |
| $\chi^2_1$ for trend | | | 0.22; $P = 0.64$ | |
| Mouthwash use (times/week) | | | | |
| Never | 152 | 128 | 1[c] | |
| 1–2 | 28 | 38 | 0.69 | (0.36–1.32) |
| ≥3 | 12 | 34 | 0.26 | (0.12–0.59) |
| $\chi^2_1$ for trend | | | 10.31; $P = 0.001$ | |
| Years with dentures | | | | |
| Never | 66 | 75 | 1[c] | |
| <10 years | 20 | 26 | 0.62 | (0.28–1.35) |
| ≥ 10 years | 114 | 99 | 1.09 | (0.64–1.86) |
| $\chi^2_1$ for trend | | | 0.16; $P = 0.69$ | |
| Dental check-ups | | | | |
| Never | 97 | 74 | 1[c] | |
| ≥ once every 5 years | 49 | 34 | 1.61 | (0.83–3.07) |
| < once every 5 years | 52 | 92 | 0.71 | (0.64–1.86) |
| $\chi^2_1$ for trend | | | 1.40; $P = 0.24$ | |
| *Interviewer-reported*: | | | | |
| Missing teeth | | | | |
| ≤5 | 14 | 39 | 1[c] | |
| 6–15 | 38 | 58 | 1.82 | (0.76–4.35) |
| ≥16 | 148 | 103 | 2.74 | (1.23–6.12) |
| $\chi^2_1$ for trend | | | 6.68; $P = 0.01$ | |
| General oral condition | | | | |
| Good | 27 | 49 | 1[c] | |
| Average | 89 | 97 | 1.82 | (0.94–3.53) |
| Poor | 82 | 49 | 2.55 | (1.24–5.24) |
| $\chi^2_1$ for trend | | | 6.21; $P < 0.02$ | |

[a]Some strata do not add up to the total because of missing values. [b]Estimates from unconditional logistic regression equations, including terms for gender, age, area of residence, education, smoking and drinking habits. [c]Reference category.

substantially since 1970 (Corrao et al, 2000) when it was the third highest worldwide after Cyprus and Greece (IARC, 1986). ORs for different levels of cigarette smoking were comparable with those reported from studies in Europe (Franceschi et al, 1999), North America (Blot et al, 1999) and Latin America (Franco et al, 1989; De Stefani et al, 1999; Hayes et al, 1999). The vast majority of smokers in Cuba had used black tobacco and unfiltered cigarettes exclusively, hence less marked increases in risk among smokers of blonde tobacco or filtered cigarettes are difficult to evaluate (Boffetta, 1993). A few heavy smokers of cigars or pipe only were found and their risk level approximately corresponded to smoking 30 cigarettes per day or more. 19% of oral cancers were attributable in Cuba to cigars or pipe. Smoking cigars only was associated with an OR for oral cancer of 7.9 in the United States (Shanks and Burns, 1998) and 9.0 in Italy and Switzerland (La Vecchia et al, 1998) (i.e., a risk intermediate between heavy and very heavy cigarette smokers). A decline in risk manifested itself in our present investigation only 10 years or more after smoking cessation, i.e., later than was found in a few larger studies on cancer of the oral cavity and pharynx (Blot et al, 1988; La Vecchia et al, 1999a; Schlecht et al, 1999). A transient risk increase 1 to 9 years after stopping was also found in a case–control study on oral cancer in Puerto Rico and suggests that a few cases may have quitted smoking for early cancer symptoms or signs.

In respect to alcohol drinking, the ORs were in agreement with findings from previous European (Franceschi et al, 1999) and North (Blot et al, 1988) and Latin American (Franco et al, 1989; Hayes et al, 1999) studies, even though the dose–response curve was markedly J-shaped. An OR above 100 emerged for the combination of the highest levels of smoking and drinking in the Cuban population. More than 70% of alcohol intake was derived from hard liquors, for which a 6-fold risk increase was found among heavy drinkers. For beer and, especially, wine, intakes were on average low-to-moderate and they corresponded to modest non-significant risk elevations. After mutual adjustment for the intake of the 3 major types of alcoholic beverages, however, ORs at each consumption level did not differ. This provides further evidence that it is ethanol, the main component of alcoholic beverages, that determines the risk of cancer and that the most frequently

© 2001 Cancer Research Campaign 

**Table 7** Odds ratios (OR) and corresponding 95% confidence intervals (CI) for cancer of the oral cavity and oro-pharynx according to selected infectious diseases and sexual habits: 200 cases and 200 controls[a]. Cuba, 1996–1999

| | No. Cases | No. Controls | OR[b] | (95% CI) |
|---|---|---|---|---|
| Warts[d] | | | | |
| Never | 122 | 102 | 1[d] | |
| Hands and feet | 16 | 17 | 1.04 | (0.43–2.50) |
| Other sites | 70 | 89 | 0.72 | (0.44–1.18) |
| Herpetic lesions[c] | | | | |
| Never | 186 | 158 | 1[d] | |
| Lip | 1 | 7 | 0.36 | (0.17–0.75) |
| Genitals and others | 13 | 36 | | |
| Candidiasis[c] | | | | |
| Never | 152 | 136 | 1[d] | |
| Mouth | 1 | 2 | 0.78 | (0.46–1.31) |
| Other sites | 48 | 64 | | |
| Gonorrhoea | | | | |
| Never | 180 | 175 | 1[d] | |
| Ever | 20 | 25 | 0.79 | (0.38–1.66) |
| Lifetime no of sexual partners | | | | |
| ≤1 | 50 | 49 | 1[d] | |
| 2–5 | 34 | 30 | 0.88 | (0.40–1.95) |
| 6–10 | 27 | 26 | 0.60 | (0.22–1.67) |
| ≥11 | 43 | 62 | 0.51 | (0.20–1.29) |
| $\chi^2_1$ for trend | | | 2.51; $P = 0.11$ | |
| Number of marriages | | | | |
| ≤1 | 119 | 103 | 1[d] | |
| 2 | 40 | 45 | 0.73 | (0.41–1.31) |
| ≥3 | 41 | 52 | 0.64 | (0.35–1.17) |
| $\chi^2_1$ for trend | | | 2.39; $P = 0.12$ | |
| Age at the first marriage | | | | |
| <20 | 67 | 70 | 1[d] | |
| 20–24 | 63 | 69 | 1.14 | (0.64–2.01) |
| ≥25 | 70 | 61 | 1.44 | (0.79–2.64) |
| $\chi^2_1$ for trend | | | 1.39; $P = 0.24$ | |
| Practice of oral sex | | | | |
| Never | 67 | 63 | 1[d] | |
| Occasionally | 26 | 40 | 0.60 | (0.29–1.23) |
| Often/most of the time | 45 | 50 | 0.80 | (0.42–1.53) |
| $\chi^2_1$ for trend | | | 0.54; $P = 0.46$ | |
| Lifetime *n* of prostitutes[e] | | | | |
| 0 | 48 | 43 | 1[d] | |
| 1–5 | 65 | 51 | 0.81 | (0.33–1.98) |
| ≥6 | 15 | 20 | 0.45 | (0.18–1.11) |
| $\chi^2_1$ for trend | | | 4.33; $P < 0.05$ | |

[a]Some strata do not add up to the total because of missing values. [b]Estimates from unconditional logistic regression equations, including terms for gender, age, area of residence, education, smoking and drinking habits. [c]Infection sites are not mutually exclusive. [d]Reference category. [e]Only males.

consumed beverage in each area (e.g., spirits in Northern Europe, Grønbæk et al, 1998; wine in Southern Europe, La Vecchia et al, 1999b; beer in Danish brewery workers; Jensen, 1979; etc.) tends to be the one with the highest risk. Former drinkers showed a steady decline in oral cancer risk (OR = 0.7 and 0.3, after 1–9 and ≥ 10 years after cessation, respectively). In contradiction to the findings for former smokers, those for former drinkers well reflect risk decline after quitting the habit for reasons unrelated to health problems (i.e., rise of alcoholic beverage prices in Cuba in the 1990s).

The interpretation of the weak associations we found with dietary habits is more difficult, since only a limited number of foods and food groups, the same for all countries participating in the international study (Herrero et al, 2000), was explored. Moderate inverse associations emerged for fruit and vegetable intake and direct ones for meat, in particular cured or salted meat, and starchy foods. Such a risk pattern is consistent with previous findings from more affluent areas of Europe (Estève et al, 1996; Levi et al, 1998; Franceschi et al, 1999) and North America (McLaughlin et al, 1988; Marshall et al, 1992). Associations between different types of meat and oral cancer have also been reported in Latin American countries (Franco et al, 1989; De Stefani et al, 1999). It is not clear whether the meat-related risk should be attributed to high salt or nitrite content, or to high saturated fat intake (Franceschi et al, 1999). The significant trend of risk increase with the increasing consumption of maize dishes is also of specific interest, since it replicated findings for upper aero-digestive tract cancer in Italy (Franceschi et al, 1990), China (Li et al, 1989); and South Africa (Van Rensburg et al, 1985). Explanations for this finding include specific nutritional deficiencies in maize-eating populations, thermal injury, and/or a role of fungal contamination of maize (e.g., fumonisins; Sydenham et al,

 *© 2001 Cancer Research Campaign*

1990). It is, however, likely that: (1) worsening of the Cuban diet in the years prior to the present study (and, hence, difficult recall of lifetime habits); (2) relatively narrow ranges of variation; and (3) lack of information on a few locally important items (e.g., tropical fruit) have weakened apparent associations with dietary habits and the corresponding attributable risk fraction.

Poor oral hygiene and number of missing teeth have been postulated as risk factors for oral cancer in many studies, including a few where smoking and drinking habits were allowed for (Marshall et al, 1992; Bundgaard et al, 1995; Velly et al, 1998; Talamini et al, 2000). In our present study, most indicators of poor oral hygiene and dentition, though not denture use, were weakly associated with increases in oral cancer risk. The strongest protection was found in regular mouth wash, although we do not have details about the specific products used. Strong associations were found for interviewer-reported variables. Oral inspection revealed that 75% of cases and 50% of controls had 16 missing teeth or more and 40% and 25%, respectively, had a 'poor general oral condition'. Each of these factors led to a more than 2.5-fold increased risk of oral cancer, but risk estimates might have been exaggerated by interviewer awareness of case identities.

Two case–control studies from the United States (Maden et al, 1992; Schwartz et al, 1998), but not one from Italy (Talamini et al, 2000), have suggested that oral cancer risk increases with the number of sexual partners and history of genital warts in men. Report of HPV-DNA in a proportion of oral cancer biopsies (Franceschi et al, 1996; Schwartz et al, 1998; Gillison et al, 2000) pointed further to a possible role of sexual habits. Despite nearly half of control subjects in Cuba reported 6 sexual partners or more and 2 or more marriages, no relation between indicators of sexual activity and oral cancer risk was found. Oral sex also seemed non-influential whereas the number of sexual partners who were prostitutes, among men, showed a significant inverse association with risk. Education and smoking and drinking habits had been carefully allowed for, but it is possible that access to prostitutes was an unspecific indicator of affluence. Indeed, low education of the patient and of the patient's spouse, and employment in manual occupations (industry or farming) approximately doubled the risk of oral cancer in Cuba. Whites were, if anything, at higher risk of oral cancer than blacks or mulattos. While the distinction between ethnic groups may be difficult in a multiethnic population like that of Cuba, our findings are in contrast with those from the United States, where oral cancer incidence is 65% higher in blacks than whites and available indicators of exposure to alcohol, tobacco and diet accounted for most, but not all, the racial differences (Day et al, 1993).

In conclusion, our present case–control study points to a decrease in very high levels of cigarette and cigar smoking as the key to oral cancer prevention in Cuba. Although alcohol drinking and dietary habits now represent relatively minor sources of risk variation, they may acquire greater unfavourable or favourable potential in the future, if the economic situation in Cuba improves. The frequency of poor dentition and the large proportion of advanced tumours (nearly half at stage 4, despite an ongoing oral cancer screening; Fernandez-Garrote et al, 1995) underline the need for strengthening programs of dental care and early diagnosis of oral cancer.

### ACKNOWLEDGEMENTS

The authors wish to thank the Pan American Health Organization (PAHO) for its generous financial contribution, and Mrs A Arslan and H Lorenzen for technical assistance.

### REFERENCES

Blot WJ, McLaughlin JK, Winn DM, Austin DF, Greenberg RS, Preston-Martin S, et al (1988) Smoking and drinking in relation to oral and pharyngeal cancer. *Cancer Res* **48**: 3282–3287

Boffetta P (1993) Black (air-cured) and blond (flue-cured) tobacco and cancer risk. V: oral cavity cancer. *Eur J Cancer* **29A**: 1331–1335

Bundgaard T, Wildt J, Frydenberg M, Elbrønd O and Nielsen JE (1995) Case-control study of squamous cell cancer of the oral cavity in Denmark. *Cancer Causes Control* **6**: 57–67

Corrao MA, Guindon GE, Sharma N and Shokoohi DF (2000) *Tobacco control country profiles*. American Cancer Society: Atlanta

Day GL, Blot WJ, Austin DF, Bernstein L, Greenberg RS, Preston-Martin S, et al (1993) Racial differences in risk of oral and pharyngeal cancer: alcohol, tobacco, and other determinants. *J Natl Cancer Inst* **85**: 465–473

De Stefani E, Deneo-Pellegrini H, Mendilaharsu M and Ronco A (1999) Diet and risk of cancer of the upper aerodigestive tract. I. Foods. *Oral Oncol* **35**: 17–21

Estève J, Riboli E, Péquignot G, Terracini B, Merletti F, Crosignani P, et al (1996) Diet and cancers of the larynx and hypopharynx: the IARC multi-center study in southwestern Europe. *Cancer Causes Control* **7**: 240–252

Fernandez Garrote L, Sankaranarayanan R, Lence Anta JJ, Rodriguez Salva A and Parkin DM (1995) An evaluation of the oral cancer control program in Cuba. *Epidemiol* **6**: 428–431

Franceschi S, Bidoli E, Baron AE and La Vecchia C (1990) Maize and risk of cancers of the oral cavity, pharynx, and esophagus in northeastern Italy. *J Natl Cancer Inst* **82**: 1407–1411

Franceschi S, Muñoz N, Bosch FX, Snijders PJF and Walboomers JMM (1996) Human papillomavirus and cancers of the upper aerodigetive tract: a review of epidemiological and experimental evidence. *Cancer Epidemiol Biom & Prev* **5**: 567–575

Franceschi S, Favero A, Conti E, Talamini R, Volpe R, Negri E, et al (1999) Food groups, oils and butter, and cancer of the oral cavity and pharynx. *Br J Cancer* **80**: 614–620

Franceschi S, Bidoli E, Herrero R and Muñoz N (2000) Comparison of the oral cavity and pharynx worldwide: etiological clues. *Oral Oncol* **36**: 106–115

Franco EL, Kowalski LP, Oliveira BV, Curado MP, Pereira RN, Silva ME, et al (1989) Risk factors for oral cancer in Brazil: a case-control study. *Int J Cancer* **43**: 992–1000

Gillison ML, Koch WM, Capone RB, Spafford M, Westra WH, Wu L, et al (2000) Evidence for a causal association between human papillomavirus and a subset of head and neck cancers. *J Natl Cancer Inst* **92**: 709–720

Grønbæk M, Becker U, Johansen D, Tønnesen H, Jensen G and Sørensen TIA (1998) Population based cohort study of the association between alcohol intake and cancer of the upper digestive tract. *Br Med J* **317**: 844–848

Hayes RB, Bravo-Otero E, Kleinman DV, Brown LM, Fraumeni JF Jr, Harty LC and Winn DM (1999) Tobacco and alcohol use and oral cancer in Puerto Rico. *Cancer Causes & Control* **10**: 27–33

Herrero R, Muñoz N, Franceschi S, Castellsagué X, Meijer CJLM, Walboomers JMM, et al (2000) International case-control study of HPV and cancer of the oral cavity and pharynx. Abstract No 166, Proceeding of the 18th International Papillomavirus Conference, Barcelona, 23–28 July 2000, p. 184

IARC (1986) Monographs on the Evaluation of the Carcinogenic Risks to Humans, Volume 38, Tobacco Smoking. International Agency for Research on Cancer: Lyon

IARC (1988) Monographs on the Evaluation of the Carcinogenic Risks to Humans, Volume 44, Alcohol Drinking. International Agency for Research on Cancer: Lyon

Jensen OM (1979) Cancer morbidity and causes of death among Danish brewery workers. *Int J Cancer* **23**: 454–463

La Vecchia C, Bosetti C, Negri E, Levi F and Franceschi S (1998) Cigar smoking and cancers of the upper digestive tract [letter]. *J Natl Cancer Inst* **90**: 1670

© 2001 Cancer Research Campaign

La Vecchia C, Franceschi S, Bosetti C, Levi F, Talamini R and Negri E (1999a) Time since stopping smoking and the risk of oral and pharyngeal cancers. *J Natl Cancer Inst* **91**: 726–727

La Vecchia C, Franceschi S, Favero A, Talamini R and Negri E (1999b) Alcohol intake and cancer of the upper digestive tract. Pattern of risk in Italy is different from that in Denmark. *Br Med J* **318**: 1289–1290

Levi F, Pasche C, La Vecchia C, Lucchini F, Franceschi S and Monnier P (1998) Food groups and risk of oral and pharyngeal cancer. *Int J Cancer* **77**: 705–709

Li J-Y, Ershow AG, Chen Z-J, Wacholder S, Li G-Y, Guo W, et al (1989) A case-control study of cancer of the esophagus and gastric cardia in Linxian. *Int J Cancer* **43**: 755–761

Maden C, Beckmann AM, Thomas DB, McKnight B, Sherman KJ, Ashley RL, et al (1992) Human papillomaviruses, herpes simplex viruses, and the risk of oral cancer in men. *Am J Epidemiol* **135**: 1093–1102

Marshall JR, Graham S, Haughey BP, Shedd D, O'Shea R, Brasure J, et al (1992) Smoking, alcohol, dentition and diet in the epidemiology of oral cancer. *Oral Oncol Eur J Cancer* **28B**: 9–15

McLaughlin JK, Gridley G, Block G, Winn DM, Preston-Martin S, Schoenberg JB et al (1988) Dietary factors in oral and pharyngeal cancer. *J Natl Cancer Inst* **80**: 1237–1243

Mezzetti M, Ferraroni M, Decarli A, La Vecchia C and Benichou J (1996) Software for attributable risk and confidence interval estimation in case-control studies. *Computers & Biomed Res* **29**: 63–75

Negri E, La Vecchia C, Franceschi S and Tavani A (1993) Attributable risk for oral cancer in Northern Italy. *Cancer Epidemiol Biom & Prev* **2**: 189–193

Parkin DM, Muir CS, Whelan SL, Gao YT, Ferlay J and Powell J (1992) Cancer Incidence in Five Continents, Vol. VI. International Agency for Research on Cancer: Lyon, 1992

Schlecht NF, Franco EL, Pintos J and Kowalski LP (1999) Effect of smoking cessation and tobacco type on the risk of cancers of the upper aero-digestive tract in Brazil. *Epidemiol* **10**: 412–418

Schwartz SM, Daling JR, Doody DR, Wipf GC, Carter JJ, Madeleine MM, et al (1998) Oral cancer risk in relation to sexual history and evidence of human papillomavirus infection. *J Natl Cancer Inst* **90**: 1626–1636

Shanks TG and Burns DM (1998) Disease consequences of cigar smoking. Smoking and tobacco control Monograph 9 Cigars. Health effects and trends. National Institutes of Health: Bethesda (MD). Report No.: DHHS Publ No. (NIH) 98–4302. pp. 105–158

Sydenham EW, Thiel PG, Marasas WPO, Shephard GS, Van Shalkwyk DJ and Kock KR (1990) Natural occurrence of some Fusarium mycotoxins in corn from low and high esophageal cancer prevalence areas of the Transkei, southern Africa. *J Agricul Food Chem* **38**: 1900–1903

Talamini R, Vaccarella S, Barbone F, Tavani A, La Vecchia C, Herrero R, Muñoz N and Franceschi S (2000) Oral hygiene, dentition, sexual habits and risk of oral cancer. *Br J Cancer* **83**: 1238–1242

Van Rensburg ES, Bradshaw D, Rose EF, et al (1985) Esophageal cancer in Zulu men, South Africa: a case-control study. *Br J Cancer* **51**: 399–405

Velly AM, Franco EL, Schlecht N, Pintos J, Kowalski LP, Oliveira BV and Curado MP (1998) Relationship between dental factors and risk of upper aerodigestive tract cancer. *Oral Oncol* **34**: 284–191

WHO (World Health Organization) (1997) Manual of the International Statistical Classification of Diseases, Injuries and Causes of Death (Based on the Recommendations of Ninth Revision Conference, 1975). World Health Organization: Geneva, pp. 141–146

*© 2001 Cancer Research Campaign*
